# Emotion Regulation as a Mechanism of Mindfulness in Individual Cognitive-Behavioral Therapy for Depression and Anxiety Disorders

**DOI:** 10.1155/2024/9081139

**Published:** 2024-04-02

**Authors:** Luise Pruessner, Christina Timm, Julia Kalmar, Hinrich Bents, Sven Barnow, Johannes Mander

**Affiliations:** ^1^Department of Psychology, Heidelberg University, Heidelberg, Germany; ^2^Center for Psychological Psychotherapy, Heidelberg University, Heidelberg, Germany

## Abstract

**Background:**

The global prevalence of depression and anxiety disorders underscores the need for a more profound comprehension of effective treatments. Mindfulness has shown promise in enhancing treatment outcomes and preventing relapse in these conditions, but the underlying mechanisms remain poorly understood.

**Methods:**

This study examined the role of emotion regulation as a mediator in the relationship between changes in mindfulness and the reduction of depression and anxiety symptoms during individual cognitive-behavioral therapy (CBT). We tracked longitudinal changes in mindfulness, emotion regulation, depression, and anxiety at baseline (pre), early treatment (session 5), midtreatment (session 15), and posttreatment (session 25) in 162 patients with depression and anxiety disorders. Further, we examined whether the effects of mindfulness on emotion regulation could be enhanced by introducing a brief mindfulness intervention at the beginning of each CBT session, as compared to progressive muscle relaxation and individual psychotherapy without any standardized session-introducing interventions.

**Results:**

Multilevel structural equation modeling indicated that decreases in rumination and increases in reappraisal and acceptance mediated the relationship between mindfulness and reductions in depressive symptoms. In contrast, reductions in avoidance explained the association between mindfulness and changes in anxiety symptoms. These links remained unchanged when adding a mindfulness intervention into individual CBT.

**Conclusion:**

Results support emotion regulatory properties of mindfulness and highlight distinct pathways of symptom reduction in depression and anxiety. These findings have important implications for understanding the mechanisms of mindfulness and tailoring treatment to individual patient needs. This trial is registered with NTC02270073.

## 1. Introduction

Depression and anxiety disorders are among the most pressing mental health concerns worldwide, with evidence showing a stable or even rising prevalence over the past several decades [1–3]. These disorders significantly contribute to the global disease burden, with anxiety disorders presenting a lifetime cumulative risk ranging from 18.3% to 31.0%, while depressive disorders exhibit a lifetime risk between 20.1% and 34.0% [4]. Beyond causing individual distress and impairment, these disorders substantially strain healthcare systems and impose extensive socioeconomic costs [5, 6]. Mitigating this global public health challenge necessitates a deeper understanding of effective treatments and relapse prevention strategies [7, 8].

Mindfulness, characterized by present-moment awareness and a nonjudgmental attitude [9–11], has emerged as a promising approach to addressing these disorders. By integrating mindfulness into manualized group treatments such as *mindfulness-based stress reduction* (MBSR) and *mindfulness-based cognitive therapy* (MBCT), there is potential for not only treating individuals afflicted with depression and anxiety disorders but also preventing relapse in these conditions [12–18]. Nevertheless, despite these promising outcomes, the precise mechanisms through which mindfulness influences depression and anxiety symptoms remain elusive [19–21].

One hypothesized explanation for the beneficial clinical effects of mindfulness on depression and anxiety has been through improving the successful use of emotion regulation strategies [22–24]. Emotion regulation strategies are commonly conceptualized as describing various ways through which individuals attempt to influence the intensity, frequency, and duration of their affective responses [25, 26]. Difficulties in emotion regulation strategy use play a central role in the development, maintenance, and treatment of depression and anxiety disorders [27–29].

A growing body of theoretical articles and empirical studies suggests a connection between mindfulness training and the enhancement of emotion regulation strategies [30–34]. These studies propose that emotion regulation strategies mediate the link between mindfulness and symptoms of depression and anxiety across both nonclinical populations [35–40] and clinical groups, including patients with social anxiety disorder [41, 42]. Furthermore, studies exploring individual differences have found that trait mindfulness and emotion regulation strategies are both uniquely and jointly associated with the severity of symptoms in patients with depression and anxiety disorders [22, 23, 43].

However, while these studies indicate a link between mindfulness, emotion regulation, and symptom severity, a lack of longitudinal studies in clinical settings restricts conclusions about these associations' nature and temporal progression [31]. Therefore, it remains unclear if emotion regulation indeed changes due to increases in mindfulness, which, in turn, is associated with reductions in clinical symptomatology. To increase the degree of causal specificity, studies that include multiple measurements of the proposed mediators, predictors and outcomes across treatment are required [31, 44, 45].

Furthermore, research on the mechanisms of mindfulness has predominantly focused on manualized group settings [12, 20, 21]. These settings incorporate many components contributing to their observed clinical benefits [46–49]. Consequently, it is crucial to examine the effects of individual components within mindfulness-based interventions to identify the underlying mechanisms of change [20, 48]. Integrating mindfulness interventions into individual psychotherapy is a core notion of third-wave cognitive-behavioral therapy (CBT) approaches [50, 51]. Nevertheless, a significant gap persists in understanding the effects of mindfulness interventions on therapeutic outcomes when embedded in individual CBT sessions [46, 49, 51].

Previous research, including our own [52, 53], suggests that mindfulness levels naturally increase during individual CBT, whether explicitly included or not. However, the role of added mindfulness in enhancing emotion regulation strategies and whether these strategies mediate the relationship between mindfulness and treatment outcomes remains unclear [48, 52]. Understanding these underlying mechanisms is crucial, as it could inform the refinement of interventions to better accommodate the dynamic processes of change that unfold over time [54, 55].

To fill these gaps in our understanding of the mechanisms of mindfulness, the present longitudinal study was designed to track the temporal progression of the proposed mediators, predictors, and treatment outcomes [44, 56, 57]. Therefore, we assessed changes in mindfulness, emotion regulation strategies, and symptom severity before treatment, during early treatment, midtreatment, and after treatment. Moreover, to overcome the limitation that mechanisms of mindfulness are mainly studied in manualized group settings, we investigated the mechanisms of mindfulness in individual CBT for depression and anxiety disorders. While our previous investigation [52] examined the effects of session-introducing mindfulness on primary treatment outcomes, the current study focused on the mediating emotion regulatory mechanisms explaining symptom changes. Specifically, we were interested in testing whether the effects of mindfulness on symptoms of depression and anxiety can be explained through two distinct emotion regulatory pathways.

The first pathway to mitigating anxiety and depression symptoms involves reducing perseverative and avoidant emotion regulation strategies, such as rumination, avoidance, and suppression, which are known to maintain and exacerbate internalizing psychopathology [29, 58–62]. Rumination is characterized by repetitive, negative, and self-focused thoughts about the causes and consequences of past experiences, leading to a cycle of negative contemplation about one's distressing emotions [58, 63, 64]. By contrast, the present-moment focus and the non-evaluative attitude inherent in mindfulness are antithetical to the abstract self-critical thinking characteristic of rumination [33, 65–69]. Similarly, mindfulness may help individuals tolerate and approach their emotions, thoughts, and bodily sensations more openly, reducing the conscious or unconscious efforts to escape from negative experiences through avoidance and suppression [70–74]. This may interrupt the feedback loop that amplifies anxiety and depression [30, 58–60].

The second pathway includes cultivating a more accepting and flexible emotion regulation approach that may protect against depression and anxiety through reappraisal, acceptance, and problem-solving [23, 34, 75–78]. Mindfulness practice promotes a decentered perspective wherein individuals observe their thoughts and feelings as transient mental events rather than reflections of the self or reality [79, 80]. According to the mindfulness-to-meaning theory [81, 82], this observational stance fosters an expanded attentional space, facilitating reframing situations from novel perspectives through reappraisal [34]. Moreover, mindfulness may enhance acceptance by promoting a nonjudgmental and compassionate attitude towards one's experiences [18, 83–85]. By learning to acknowledge emotions without criticism or resistance, individuals can diminish the intensity and associated distress of their experiences [85]. Finally, the increased awareness promoted by mindfulness may allow individuals to maintain attention on a task and encourage them to look at the bigger picture rather than get stuck on specific details of a particular problem [86, 87]. This focused attention and heightened cognitive flexibility may benefit problem-solving, allowing for the generation of creative solutions [77, 78, 86, 87].

To empirically test these two hypothesized pathways, the present study examined mediational models in which changes in emotion regulation were proposed to explain the relationship between increases in mindfulness and reductions in symptoms of depression and anxiety throughout individual CBT treatment. Consistent with the presented theory and research, we predicted that the links between increases in mindfulness and changes in depression and anxiety would be mediated by reductions in rumination, suppression, and avoidance [37, 65, 70]. Furthermore, we expected that the employment of strategies such as reappraisal, acceptance, and problem-solving would increase following elevated mindfulness, which, in turn, was hypothesized to explain symptom reductions [81, 85, 86]. Finally, we aimed to test whether these effects of mindfulness on emotion regulation could be enhanced by including a mindfulness intervention in individual psychotherapy. Specifically, we tested whether there are more substantial improvements in emotion regulation strategies if mindfulness is included as a standardized session-introducing intervention in each CBT session compared to conditions of progressive muscle relaxation and individual psychotherapy without any standardized session-introducing interventions.

## 2. Method

### 2.1. Participants

One hundred sixty-two participants with depression or anxiety disorders were recruited at a large outpatient treatment center in Germany. This sample size was determined based on power analyses to detect small effects (Cohen's *f* = 0.12; for details, see [47, 52]). To maximize our findings' external validity and clinical applicability, inclusion criteria were broad and comprised an age between 18 and 65 years and a primary depressive disorder or an anxiety disorder diagnosis based on the *Structured Clinical Interview* according to the DSM-5 criteria (SCID-5) [88, 89]. To be eligible for the study, all potential participants underwent initial phone interviews capturing the presence of depressive and anxiety symptoms. If these preliminary responses indicated a clinical depression or anxiety disorder, individuals were invited to a face-to-face SCID-5 interview. All interviews were recorded on video, and 20% were rated by a second clinician (*N* = 33), yielding a high interrater reliability (*κ* = 0.93, *p* < 0.001). Exclusion criteria were (a) insufficient German language skills, (b) diagnosis of a psychotic disorder, (c) acute suicidality, and (d) severe medical illnesses (e.g., cancer) that might interfere with participating in a study.

Among individuals primarily diagnosed with anxiety disorders, the majority of cases were attributed to social anxiety disorder (43.5%) and panic disorder (30.4%). The most frequent comorbidities within this group were depressive disorders, observed in 46.9% of cases. In contrast, individuals with a primary diagnosis of depression predominantly presented with a current major depressive episode (78.5%), with a substantial subset experiencing recurrent episodes and currently manifesting moderate severity (45.2%). The prevalence of comorbid conditions among these individuals included personality disorders (7.7%), substance use disorders (7.7%), and eating disorders (5.1%). Further delineation of the sample's demographic and clinical characteristics is provided in Table 1.

### 2.2. Procedure

The current randomized controlled trial was registered at *ClinicalTrials.gov* (NTC02270073) and followed the CONSORT statement (for a flow chart, see [52]). The trial's primary objective was to investigate the clinical benefits of incorporating a session-introducing mindfulness intervention into individual CBT for depression and anxiety disorders [47, 52]. All participants who met the inclusion criteria received standard individual CBT for depression and anxiety disorders and were randomly allocated to one of three session introduction conditions: (1) a five-minute standardized mindfulness intervention at the beginning of every therapy session (TAU + M), (2) a five-minute standardized progressive muscle relaxation intervention (TAU + PMR), or (3) no standardized session-introducing intervention (TAU). The institutional review board at Heidelberg University approved the trial following the Helsinki Declaration.

### 2.3. Intervention Conditions

In the *TAU + M* condition, each session started with a five-minute adapted version of the breathing space from the MBCT program [90]. This intervention was audiotaped and guided by a trained mindfulness expert. Participants started by focusing on their breath to anchor them in the present moment. Subsequently, they broadened their awareness to include sensations throughout the body, cultivating an attitude of acceptance and openness (for detailed instructions, see [52]).

In the *TAU + PMR* condition (active control), a five-minute audiotaped version of the PMR exercise was conducted at the beginning of the therapy session. The PMR technique used in this study followed the standard protocol outlined by Jacobson adapted for individual psychotherapy [91] and involved the systematic tensing and relaxing of muscle groups. PMR differs from mindfulness by offering physiological feedback and structured guidance tailored to prompt immediate physical relief. Its primary objective is to cultivate a deep state of relaxation, contrasting with the mindfulness goal of fostering a nonreactive, observant state [92].

If patients were randomized into the *TAU* condition (passive control), they received a CBT treatment without standardized session-introducing interventions related to mindfulness or progressive muscle relaxation at the beginning of the therapy sessions. Instead, therapists could initiate each therapy session with an individualized agenda following standard CBT protocols for depression and anxiety disorders.

While the group assignment specified the initial session activity, therapists across the TAU + M, TAU + PMR, and TAU groups had the flexibility to tailor the treatment plans to the specific needs of their patients with depression and anxiety disorders. This individualization could involve integrating mindfulness practices into standard CBT protocols, ensuring that the treatment adheres to routine care standards without restricting the incorporation of beneficial practices.

### 2.4. Measures

To address the methodological requirements for investigating mechanisms of change [44, 45], patients completed measures of mindfulness, emotion regulation, and symptom severity at baseline (pre), the early treatment stage (session 5), the midtreatment stage (session 15), and the end of treatment (session 25).

#### 2.4.1. Mindfulness

The patient's development of mindfulness skills throughout CBT was measured using the *Kentucky Inventory of Mindfulness Skills* (KIMS) [93, 94]. This inventory comprises 39 items evaluated on a five-point scale (e.g., “I notice changes in my body, such as whether my breathing slows down or speeds up.” and “When I'm doing something, I'm only focused on what I'm doing, nothing else.”) and has been validated in various settings. In the current study, internal consistency was between 0.81 and 0.90.

#### 2.4.2. Emotion Regulation

Changes in emotion regulation strategies were assessed using the *Heidelberg Form for Emotion Regulation Strategies* (HFERST) [95]. It consists of 28 items rated on a five-point scale capturing reappraisal (e.g., “I change my feelings by thinking about the situation differently.”), acceptance (e.g., “When change is impossible, I accept the situation as it is.”), problem-solving (e.g., “I consider how to best handle the situation when encountering problems.”), rumination (e.g., “I find myself dwelling on the reasons behind my negative emotions.”), suppression (e.g., expressive: “It is hard for other people to tell how I am feeling.”; experiential: “When I have strong emotions, I immediately push them aside.”), and avoidance (“I prefer to avoid situations that could elicit negative emotions.”). The measure has provided evidence for its reliability and validity [95], and internal consistency in our sample ranged from 0.79 to 0.87.

#### 2.4.3. Depressive Symptoms

Depressive symptoms were assessed using the *Beck Depression Inventory* (BDI-II) [96, 97], which comprises 21 items rated on a four-point scale (e.g., “I have lost most of my interest in other people or things.” and “I feel my future is hopeless and will only get worse.”). The BDI-II has excellent psychometric properties [96, 97]. In our current study, internal consistency was within the range of 0.91 and 0.95.

#### 2.4.4. Anxiety Symptoms

Anxiety symptoms were captured based on the *Beck Anxiety Inventory* (BAI) [98, 99]. This questionnaire consists of 21 items rated on a four-point scale (e.g., being bothered by “fear of worst happening,” “fear of losing control,” feeling “terrified or afraid,” “scared,” or “nervous”) and has demonstrated strong reliability and validity [98, 99]. Internal consistency in our sample was between 0.90 and 0.92.

### 2.5. Statistical Analyses

The mediating effects of emotion regulation strategies on the longitudinal associations between mindfulness and symptom severity were tested based on multilevel structural equation modeling (MSEM) in the R environment using the package “lavaan” [100]. The choice of MSEM was driven by the hierarchical data structure, where measurement time (level 1; within-person effects) was nested within individuals (level 2; between-person effects) [101–103]. Moreover, this approach helps mitigate estimation bias concerning the indirect effect and allows for the differentiation between within-person and between-person components of the indirect effect [104, 105].

Within this analytical framework, both within-person mediation and between-person mediation were examined using mindfulness as a predictor variable, emotion regulation strategies as mediators, and depression or anxiety as outcome variables. To mitigate the potential for type 1 errors [101, 104], we specified multiple mediation models. These models focused on strategies hypothesized to be reduced following elevated mindfulness (i.e., rumination, suppression, and avoidance) in contrast to strategies expected to increase as a result of heightened mindfulness (i.e., reappraisal, acceptance, and problem-solving) and their distinct roles in predicting depression versus anxiety.

On the within-person level, the analyses were run as 1-1-1 mediation models with measures regressed on baseline scores to examine all variables as latent change scores, respectively [101–103]. This allowed us to capture changes in these constructs within individuals across time. On the between-person level, individual differences in the average levels of mindfulness, emotion regulation strategies, and depression or anxiety were explored. Moreover, the study condition (i.e., TAU + M, TAU + PMR, and TAU) was included as a between-person level predictor to test whether the type of session introduction impacts the use of emotion regulation strategies.

In the mediational models, total effects were assessed to determine the magnitude of the impact of changes in mindfulness on depression and anxiety symptoms (*c*-path). Subsequently, we estimated the direct effects of mindfulness on emotion regulation strategies (*a*-path) and the direct effects of emotion regulation strategies on depression and anxiety (*b*-path).

The indirect effect of mindfulness on depression or anxiety through emotion regulation strategies was calculated as the product of the *c*-path and *b*-path (*ab*-path), testing whether the associations between mindfulness and symptom severity could be explained by higher reappraisal, acceptance, and problem-solving, or reduced use of rumination, suppression, and avoidance.

Model parameters were estimated using robust maximum likelihood estimation to handle non-normality in the data. Model fit was assessed using the Comparative Fit Index (CFI), Tucker-Lewis Index (TLI), root mean square error of approximation (RMSEA), and standardized root mean square residual (SRMR). Adequate fit was indicated by CFI/TLI values close to 0.95, RMSEA values close to or below 0.06, and SRMR values close to or below 0.08 [106].

Before these primary analyses, the presence of within-person temporal change in emotion regulation, mindfulness, and symptom severity was examined using two-level multilevel growth models [107–109] within the R package “lme4” [110]. The initial model incorporated random intercepts at the between-person level without any predictors. The subsequent model introduced both fixed and random effects of time. Third, the covariates of age, gender, and diagnosis were added as potentially relevant moderators. Finally, we included the time-by-condition interaction effects (TAU + M, TAU + PMR, and TAU) to assess whether the session-introductory mindfulness intervention induced more substantial alterations in emotion regulation than the other conditions. We employed likelihood ratio tests to evaluate model fit and calculated the Akaike information criterion [111]. The magnitude of temporal changes was assessed using Cohen's *d* effect size for multilevel models based on the *lme.dscore* command in R [112]. Moreover, within- and between-person correlations were explored using the *statsBy* function in the package “psych” [113].

## 3. Results

### 3.1. Descriptive Statistics and Temporal Changes

Table 2 presents the means and standard deviations of the key study variables over time, providing an overview of their temporal changes. The results from multilevel growth models, which analyzed the trajectories of change following treatment, revealed significant shifts in these variables. Specifically, mindfulness, reappraisal, acceptance, and problem-solving all exhibited notable increases throughout the course of CBT (all *p* < 0.017; Table 2). Conversely, rumination, suppression, avoidance, depression, and anxiety showed a temporal decrease (all *p* < 0.001). The effect sizes of these within-person changes were medium to large concerning heightened levels of mindfulness, reappraisal, and acceptance (all *d* ≥ 0.57 [0.25, 0.89]) and small for increases in problem-solving (*d* = 0.24 [0.01, 0.48]). Effect sizes for reductions in rumination, depression, and anxiety were large (all *d* ≥ −0.71 [-1.03, -0.38]), while decreases in avoidance and suppression were medium-sized (*d* ≥ −0.48 [-0.79, -0.16]).

As a prerequisite for the primary analyses, the within- and between-person correlations between all study measures are shown in Table 3. Notably, mindfulness skills were linked to lower symptoms of depression and anxiety, as well as a higher propensity for reappraisal, acceptance, and problem-solving, and reduced utilization of rumination, suppression, and avoidance on both the within- and between-person levels (all *p* < 0.001).

### 3.2. Multilevel Structural Equation Models

**Figure 1:**
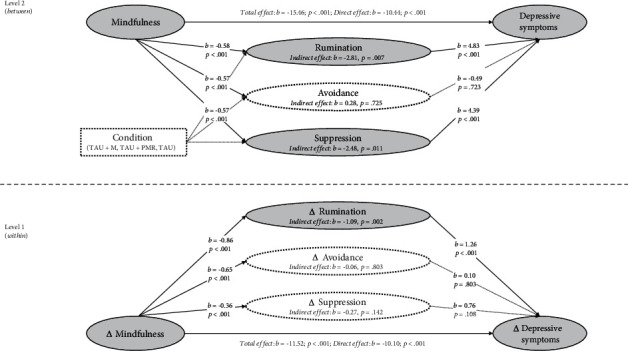
Multilevel mediation analyses with indirect effects of rumination, avoidance, and suppression on depressive symptoms.

**Figure 2:**
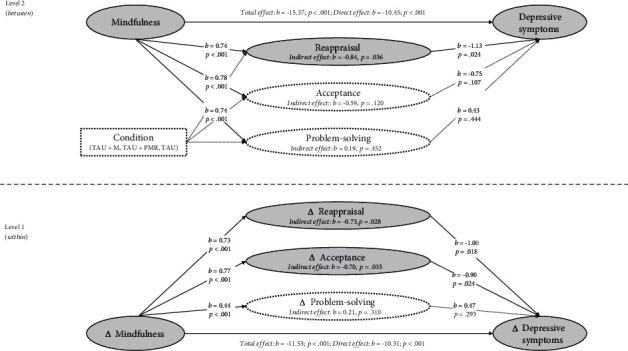
Multilevel mediation analyses with indirect effects of reappraisal, acceptance, and problem-solving on depressive symptoms.

Results of the MSEM analyses with depressive symptoms as outcomes are visually presented in Figures 1 and 2. Model fit for all within- and between-person mediational models testing the relationships between mindfulness, emotion regulation strategies, and depression was good (RMSEA ≤ 0.044; CFI ≥ 0.997; TLI ≥ 0.939; SRMR ≤ 0.019), indicating that the models adequately represented the observed data. Detailed coefficients for total, direct, and indirect effects, as well as the model fit indices for these analyses, are provided in supplementary Tables S1-S2.

At the within-person level (level 1), the total effect paths indicated that increases in mindfulness were associated with reductions in depressive symptoms (*c*-paths: *b*_1_ = −11.52, 95% CI [-13.75, -9.30]; *b*_2_ = −11.53, 95% CI [-13.76, -9.30]). Moreover, the direct effects of increases in mindfulness on emotion regulation strategies were significant for all regulatory strategies (*a*-paths: all |*b*| ≥ 0.36, all *p* < 0.001). In turn, the direct effects of emotion regulation on depressive symptoms revealed that changes in rumination, reappraisal, and acceptance were associated with reductions in depression (*b*-paths: all |*b*| ≥ 0.90, all *p* ≤ 0.024).

In our exploration of indirect effects, we found that the relationships between changes in mindfulness and depression were partially mediated by alterations in these three emotion regulation strategies. Specifically, reductions in rumination (*ab*-path: *b* = −1.09, 95% CI [-1.80, -0.39]) and increases in reappraisal (*ab*-path: *b* = −0.73, 95% CI [-1.38, -0.08]) and acceptance (*ab*-path: *b* = −0.70, 95% CI [-1.34, -0.05]) had a significant indirect effect on depressive symptoms. No notable indirect effects were observed for changes in the other emotion regulation strategies (all *p* ≥ 0.14).

Most of these findings were replicated at the between-person level (level 2). The analysis revealed that the link between mindfulness and depressive symptoms was partially mediated by rumination, suppression, and reappraisal (all |*b*| ≥ 0.84, all *p* ≤ 0.036; see Figures 1 and 2).

**Figure 3:**
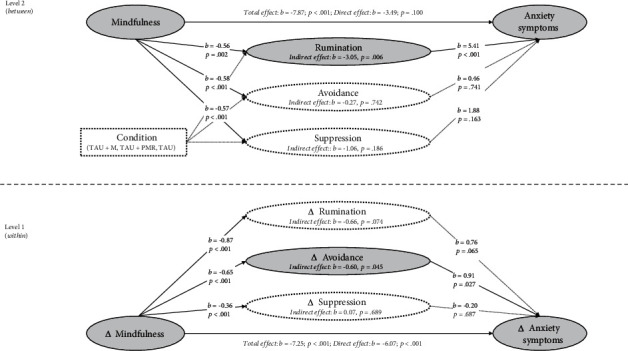
Multilevel mediation analyses with indirect effects of rumination, avoidance, and suppression on anxiety symptoms.

**Figure 4:**
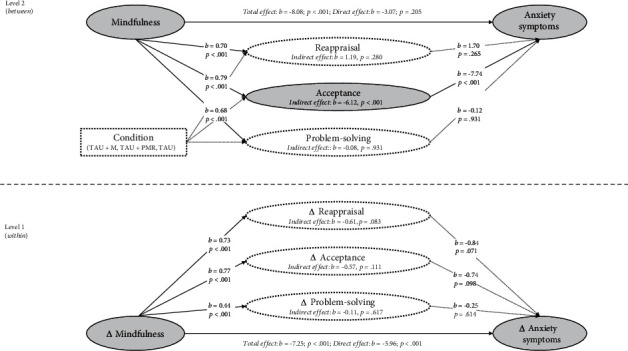
Multilevel mediation analyses with indirect effects of reappraisal, acceptance, and problem-solving on anxiety symptoms.

The MSEM analyses with anxiety symptoms as the outcomes are depicted in Figures 3 and 4. The model fit indices indicated that the analytical models adequately represented the observed data (RMSEA ≤ 0.035; CFI ≥ 0.990; TLI ≥ 0.935; SRMR ≤ 0.030). The total, direct, and indirect effects, as well as the detailed model fit indices, are shown in supplementary Tables S3-S4.

In the within-person analysis, the total effect paths revealed a negative association between increases in mindfulness and reductions in anxiety symptoms (*c*-path: *b*_1_ = −7.25, 95% CI [-10.05, -4.45]; *b*_2_ = −7.25, 95% CI [-9.78, -4.71]). Moreover, direct effects of increases in mindfulness on emotion regulation strategies were observed for all regulatory strategies (*a*-paths: all |*b*| ≥ 0.36, all *p* < 0.001). Conversely, the direct effects of emotion regulation on anxiety symptoms showed that changes in avoidance predicted reductions in anxiety (*b*-path  = −0.91, 95% CI [0.10, 1.72]). Similarly, when investigating indirect effects, the associations between changes in mindfulness and anxiety were partially mediated by reductions in avoidance (*ab*-path: *b* = −0.60, 95% CI [-1.18, -0.01]) but not by any other emotion regulation strategy (all |*b*| ≤ 0.66, all *p* ≥ 0.074).

At the between-person level, the analysis demonstrated that the relationship between mindfulness and anxiety symptoms was mediated through rumination and acceptance (all |*b*| ≥ 3.05, all *p* ≤ 0.006; see Figures 3 and 4).

### 3.3. Effects of Session-Introducing Mindfulness Practice

Finally, to examine the influence of introducing a mindfulness intervention at the beginning of each CBT session on our established models, we incorporated the study condition (TAU + M vs. TAU + PMR vs. TAU) as a person-level predictor in the MSEM and growth curve analyses. Detailed findings from these analyses are available in Tables S5-S6 in the supplementary materials.

Notably, the results of our mediation analyses remained consistent even after including the session-introducing mindfulness intervention as a predictor in the MSEM models. This suggests that the direct and indirect effects of mindfulness-related changes on reducing levels of depression and anxiety were consistent across all CBT conditions (all |*b*| ≤ 0.13; all *p* ≥ 0.243; see Table S6).

Furthermore, the growth curve analyses revealed that integrating a brief, five-minute mindfulness intervention as a session-introducing intervention was associated with a slight improvement in problem-solving as an emotion regulation strategy (*d* = 0.22, *p* = 0.029) compared to the TAU condition. Nevertheless, there were no discernible differences in the change patterns of the other emotion regulation strategies across the TAU + M, TAU + PMR, and TAU groups (all *p* ≥ 0.179, see Table S7), underscoring the consistency of our findings across the various CBT conditions. Moreover, adherence to the intervention conditions was consistently high among observer ratings, patients, and therapists. Importantly, none of the participants engaged in interventions from the nonassigned condition, affirming fidelity to the prescribed protocols [52].

## 4. Discussion

The overarching goal of the present study was to investigate emotion regulation as a potential mechanism of the relationship between increases in mindfulness and symptom reductions in individual CBT for clinical depression and anxiety disorders. Although past empirical studies indicate a link between trait mindfulness, emotion regulation strategies, and symptoms of depression and anxiety in both clinical [22, 23] and nonclinical populations [35–38], longitudinal studies examining changes in emotion regulation as a mediator between increases in mindfulness and symptom reduction during psychotherapy remain scarce. Moreover, while mindfulness-based group interventions have revealed beneficial clinical effects on depression and anxiety disorders [14–18, 78], research of specific mechanisms underlying the treatment benefits of mindfulness in individual CBT of depression and anxiety disorders is still in its early stages.

Our study offers first explanations of potential underlying mechanisms by investigating longitudinal mediation effects of emotion regulation strategies using MSEM. This method allows for distinguishing the between-person and within-person facets of change mechanisms [57]. Importantly, preliminary findings suggest that these trait-like and state-like mechanisms can exhibit contrasting correlations with treatment effectiveness [114]. Nevertheless, multilevel mediation analyses in psychotherapy research have only recently begun to appear [115]. To our knowledge, this is one of the first studies investigating emotion regulation as a mediator of mindfulness in a design that includes longitudinal measures of changes across individual CBT. Our evidence suggests that changes in emotion regulation strategies are not just consequential but are mediating how mindfulness relates to depression and anxiety over time.

Regarding the first hypothesized pathway of emotion regulation, we found that reduced rumination mediated longitudinal changes in depression, whereas reductions in avoidance mediated alterations in anxiety symptoms. The observed role of rumination corroborates existing literature, which identifies the characteristic repetitive and passive focus on distress symptoms, alongside their potential causes and consequences, as a fundamental element in perpetuating and worsening depressive symptoms [58, 65, 66, 69]. Likewise, the finding that avoidance mediates changes in anxiety symptoms aligns with prior research [61, 71]. These studies posit that avoidance, which involves escaping distressing thoughts and feelings, is a prevalent and debilitating feature in the spectrum of anxiety disorders [116]. What is particularly notable about these findings is the differential pattern in which mindfulness appears to be linked to depressive and anxiety symptoms. Although our results reveal that both strategies are associated with mindfulness and symptomatology on the between-person level, a deeper exploration into longitudinal shifts indicates differences in the enhancement of specific strategies, depending on the symptomatology [23]. This divergence highlights the importance of recognizing and addressing the unique emotion regulation profiles of patients with depression and anxiety disorders, potentially leading to improved treatment outcomes [32, 35, 37].

For the second pathway, our observation of reappraisal mediating changes in depression aligns with the theoretical premises of the mindfulness-to-meaning theory [81, 82]. Based on this framework, the decentered perspective inherent in mindfulness facilitates a shift from automatic, habitual appraisals to more adaptive and flexible interpretations. Our findings support this notion, indicating that mindfulness may prompt new appraisals in situations that typically elicit stress or threat responses. In contrast to other studies empirically testing this theoretical framework in nonclinical samples using well-being as an outcome [34, 117], we demonstrate that these assumptions can also be confirmed in a clinical sample when predicting longitudinal changes in depression. Additionally, we found that while trait-like variation in acceptance relates to mindfulness and both depression and anxiety symptoms, longitudinal increases in acceptance specifically mediated changes in depressive symptoms [85]. This evidence indicates that the effects of acceptance might vary between depressive and anxiety symptoms, being more dynamic and pronounced in the former and more static and trait-like in the latter.

Together, these findings emphasize that mindfulness can be understood as an overarching meta-approach to interacting with one's inner experiences, leading to alterations in specific emotion regulation strategies. However, contrasting theories argue that mindfulness may also be considered an emotion regulation strategy itself [30–32, 118, 119]. For example, the dual-mode model of mindful emotion regulation [119] elucidates this perspective by proposing two distinct but interconnected modes of how mindfulness operates in regulating emotions. In the *implementation mode*, mindfulness can be actively and deliberately used to target specific aspects of emotional experiences. On the other hand, the *facilitation mode* emphasizes that mindfulness, cultivated over time, inherently modifies how emotions are generated, experienced, and regulated based on various emotion regulation strategies. While the current study primarily focused on this latter, facilitative mode of mindfulness, it is imperative for future research to also explore the process of how mindfulness transitions from an active emotion regulation strategy to a more ingrained way of relating to one's internal states.

Building upon this understanding, our study further explored how CBT with and without a mindfulness add-on component alters the dynamics of mindfulness, emotion regulation, and symptom severity over time. Notably, our study revealed substantial temporal changes in mindfulness, depression, anxiety, and all assessed emotion regulation strategies in the three conditions of CBT. The observed declines in rumination, suppression, and avoidance, coupled with increases in reappraisal, acceptance, and problem-solving, underscore the malleable nature of these constructs in response to therapeutic interventions. These shifts were consistent across all experimental conditions, with the group receiving the add-on mindfulness component showing only a marginally greater enhancement in problem-solving abilities. Thus, integrating a five-minute mindfulness component into individual psychotherapy was associated with only minor additional effects on emotion regulatory processes.

The enhancements in mindfulness across all conditions might be attributed to the use of mindfulness practices within the broader CBT treatment framework. While mindfulness was not initiated at the beginning of sessions in both control groups, the engagement with other mindfulness exercises as part of regular treatment could have contributed to the overall increase observed. However, this area warrants further investigation in future studies as we did not evaluate the use of other mindfulness practices during the sessions. We examined the participants' familiarity with mindfulness at baseline and their ongoing mindfulness practices outside of the sessions at each of the four measurement points, finding that these variables did not significantly moderate the treatment effects (see [52] for detailed analyses). Additionally, the intrinsic elements of traditional CBT might have contributed to these findings. Techniques such as exposure, cognitive restructuring, and behavioral activation may inadvertently cultivate increases in mindfulness by promoting active confrontation and acceptance of distressing emotions, as well as a heightened attentiveness to internal experiences [120].

These results underscore the capacity of individual CBT not only to address specific depressive and anxiety symptoms but also to enhance mindfulness and emotion regulation, thereby confirming its multifaceted effectiveness. Yet, this realization also calls for more detailed and discerning research. Future studies need to focus on the intensity and distinct elements of mindfulness practices embedded in CBT to pinpoint their impacts and effectiveness. Given the diversity in mindfulness techniques—from breath awareness and recognition of thoughts and feelings to cultivating self-compassion—a systematic and granular examination of each component is vital [50]. Unpacking these elements will be crucial for understanding how to optimize individual CBT based on mindfulness interventions. This systematic approach is essential in advancing therapeutic methods and deepening our comprehension of the complex interplay between mindfulness and emotion regulation in the context of treating depression and anxiety disorders.

### 4.1. Strengths and Limitations of the Study

Our study used a longitudinal RCT design, thereby allowing us to formally assess whether the observed changes in emotion regulation occur throughout treatment. In doing so, our research advances upon previous cross-sectional investigations into the mechanisms of mindfulness [23, 36, 37]. It takes a step further by examining the temporal dynamics of the proposed mediators and outcome variables to investigate mediation effects. This approach provides insights into the potential mechanisms driving change, as highlighted in the literature [44, 121, 122]. Furthermore, to enhance the generalizability of our findings to real-world clinical practice, our examination of these mechanisms occurred within a naturalistic treatment setting. Notwithstanding this clinical relevance, we examined theoretically driven questions and operationalized mindfulness using controlled, experimental methods (i.e., standardized audio-recorded session-introducing interventions). Moreover, we employed robust statistical methods such as MSEM for testing within- and between-person mediation [101–103].

These strengths have to be set against the weaknesses. First, establishing emotion regulation as a longitudinal mediator of mindfulness still does not clearly identify it as a causal change mechanism. Although our longitudinal design provides more evidence for causality than cross-sectional data, the primary assumption of mediation analyses is that there is no unmeasured confounder between the mediator and outcome [105, 123, 124]. Therefore, future studies should also test for other mediating variables to consolidate the current results. Moreover, our study was conducted in a naturalistic setting in Germany, and we intentionally applied broad inclusion criteria to ensure the generalizability of our results to individuals with depression and anxiety disorders. However, due to our inclusive approach, a significant proportion of the participants reported comorbidities, which added complexity to the data. To further validate and extend the relevance of our findings, it is vital for future research to replicate this study across varied cultural and clinical contexts, offering a more nuanced understanding of these associations between mindfulness and emotion regulation. Finally, our study relied on self-report measures of metaconscious states and higher-order cognitive processes to which participants might have restricted access. Future research should adopt a multimethod framework, integrating psychotherapy research with psychophysiological methodologies. This approach should include a variety of emotion regulation measures (e.g., [125–128]) and incorporate observer-based assessments of depressive and anxiety symptoms (e.g., [129, 130]) for a more holistic understanding.

### 4.2. Conclusion and Future Directions

In sum, the current study furthers our understanding of the underlying mechanisms of mindfulness in individual CBT. Consistent with the theoretical premises of mindfulness-based psychotherapy approaches, emotion regulation strategies served as mediators of mindfulness on changes in clinical depression and anxiety. Furthermore, our investigation revealed distinct emotion regulatory pathways predicting changes in depression compared to anxiety symptoms. These findings emphasize the diverse routes through which symptoms are alleviated in individuals with depression and anxiety disorders and contribute to the expanding literature that underscores the fundamental role of emotion regulation for mindfulness [23, 36, 119]. If future research can substantiate emotion regulation as a causal mechanism of mindfulness, this would have significant implications for developing more targeted and ultimately more effective interventions for depression and anxiety disorders.

## Figures and Tables

**Table 1:** Demographic baseline characteristics of participants.

Measure	Total sample (*N* = 162)	TAU + M (*n* = 54)	TAU + PMR (*n* = 54)	TAU (*n* = 54)	*p*
Age, *M* (SD)	35.07 (12.69)	37.20 (12.47)	32.59 (12.35)	35.43 (13.05)	0.16^b^
Male, *n* (%)	64 (39.5)	18 (33.3)	21 (38.9)	25 (46.3)	0.39^c^
High school diploma, *n* (%)	72 (44.4)	22 (40.7)	27 (50.0)	23 (42.6)	0.59^b^
Completed job training, *n* (%)	100 (61.7)	39 (72.2)	31 (57.4)	30 (55.6)	0.15^b^
Status of employment (employed), *n* (%)	119 (73.5)	43 (80.0)	42 (77.8)	34 (63.0)	0.10^b^
Current relationship, *n* (%)	95 (58.6)	33 (61.1)	34 (63.0)	28 (51.9)	0.45^b^
Major diagnosis: depressive disorder, *n* (%)	93 (57.4)	32 (59.2)	31 (57.4)	30 (55.6)	0.93^b^
Major diagnosis: anxiety disorder, *n* (%)	69 (42.6)	22 (40.7)	23 (42.6)	24 (44.4)	0.93^b^
Additional comorbid diagnosis, *n* (%)	71 (43.8)	28 (51.9)	23 (42.6)	20 (37.0)	0.29^b^
Former psychotherapy, *n* (%)	91 (56.1)	32 (59.3)	28 (51.9)	31 (57.4)	0.72^b^
Pharmacotherapy, *n* (%)	77 (47.5)	23 (42.6)	24 (44.4)	30 (55.6)	0.35^b^
Familiarity with mindfulness^a^, *n* (%)	56 (34.6)	21 (38.9)	18 (33.3)	17 (31.5)	0.70^b^
Familiarity with PMR, *n* (%)	56 56 (34.6)	20 (37.0)	20 (37.0)	16 (29.6)	0.65^b^

*Note. M* = mean; SD = standard deviation; TAU+M = cognitive-behavioral therapy integrating a five-minute standardized mindfulness exercise; TAU+PMR = cognitive-behavioral therapy integrating a five-minute standardized progressive muscle relaxation exercise; TAU = cognitive-behavioral treatment without any standardized session-introducing interventions. ^a^Familiarity with mindfulness was assessed with the item “Do you have prior experience with mindfulness exercises?”. Participants with prior experience were asked about the duration, types, and frequency of their mindfulness practice. ^b^Analysis of variance. ^c^*χ*^2^ test.

**Table 2:** Means, standard deviations, and results of growth modeling analyses testing changes in mindfulness, emotion regulation, and symptom severity.

	Pre		Session 5		Session 15		Session 25		Effect size (*d*_MLM_) [95% CI]	*p*
	*M*	SD	*M*	SD	*M*	SD	*M*	SD		
Mindfulness	1.96	(0.39)	1.98	(0.44)	2.04	(0.46)	2.11	(0.49)	0.61 [0.28, 0.93]	<0.001
Rumination	4.04	(0.88)	3.74	(0.99)	3.62	(0.99)	3.48	(1.01)	-0.80 [-1.13, -0.47]	<0.001
Avoidance	3.55	(0.87)	3.35	(0.98)	3.17	(0.90)	3.14	(0.95)	-0.56 [-0.87, -0.23]	<0.001
Suppression	3.08	(0.81)	2.96	(0.83)	2.86	(0.75)	2.80	(0.74)	-0.48 [-0.79, -0.16]	<0.001
Reappraisal	2.38	(0.86)	2.58	(0.88)	2.70	(0.82)	2.74	(0.89)	0.59 [0.27, 0.91]	<0.001
Acceptance	2.55	(0.88)	2.84	(0.92)	2.81	(0.87)	2.93	(0.96)	0.57 [0.25, 0.89]	<0.001
Problem-solving	3.54	(0.90)	3.60	(0.78)	3.64	(0.78)	3.67	(0.74)	0.24 [0.01, 0.48]	0.017
Depression	26.08	(11.19)	20.48	(11.60)	18.03	(11.23)	16.19	(12.40)	-0.98 [-1.32, -0.64]	<0.001
Anxiety	18.54	(11.21)	17.13	(10.62)	15.69	(10.69)	13.88	(10.37)	-0.71 [-1.03, -0.38]	<0.001

*Note*. Effect sizes (*d*_MLM_) for growth models were calculated using R's *lme.dscore* function [112].

**Table 3:** Within- and between-person correlations between mindfulness, emotion regulation, and symptom scores with confidence intervals.

Variable	(1)	(2)	(3)	(4)	(5)	(6)	(7)	(8)	(9)
Mindfulness (1)	—	-0.33⁣^∗∗∗^ [-0.40, -0.25]	-0.23⁣^∗∗∗^ [-0.31, -0.15]	-0.18⁣^∗∗∗^ [-0.26, -0.09]	0.29⁣^∗∗∗^ [0.21, 0.37]	0.29⁣^∗∗∗^ [0.21, 0.37]	0.20⁣^∗∗∗^ [0.11, 0.28]	-0.45⁣^∗∗∗^ [-0.51, -0.38]	-0.29⁣^∗∗∗^ [-0.37, -0.21]
Rumination (2)	-0.31⁣^∗∗∗^ [-0.45, -0.16]	—	0.21⁣^∗∗∗^ [0.12, 0.29]	0.17⁣^∗∗∗^ [0.09, 0.26]	-0.21⁣^∗∗∗^ [-0.29, -0.13]	-0.22⁣^∗∗∗^ [-0.30, -0.14]	-0.06 [-0.14, 0.03]	0.28⁣^∗∗∗^ [0.20, 0.35]	0.20⁣^∗∗∗^ [0.11, 0.28]
Avoidance (3)	-0.31⁣^∗∗∗^ [-0.45, -0.16]	0.17⁣^∗^ [0.01, 0.32]	—	0.29⁣^∗∗∗^ [0.21, 0.37]	-0.17⁣^∗∗∗^ [-0.25, -0.09]	-0.11⁣^∗^ [-0.19, -0.02]	-0.07 [-0.16, 0.01]	0.23⁣^∗∗∗^ [0.15, 0.31]	0.21⁣^∗∗∗^ [0.13, 0.29]
Suppression (4)	-0.33⁣^∗∗∗^ [-0.46, -0.18]	0.11 [-0.05, 0.26]	0.37⁣^∗∗∗^ [0.23, 0.50]	—	-0.13⁣^∗∗^ [-0.21, -0.04]	-0.08 [-0.16, 0.01]	0.01 [-0.07, 0.10]	0.19⁣^∗∗∗^ [0.11, 0.27]	0.07⁣^∗^ [-0.02, 0.16]
Reappraisal (5)	0.37⁣^∗∗∗^ [0.22, 0.50]	-0.03 [-0.18, 0.13]	-0.11 [-0.26, 0.05]	0.03 [-0.13, 0.18]	—	0.32⁣^∗∗∗^ [0.24, 0.39]	0.31⁣^∗∗∗^ [0.23, 0.39]	-0.27⁣^∗∗∗^ [-0.34, -0.19]	-0.20⁣^∗^ [-0.32, -0.02]
Acceptance (6)	0.40⁣^∗∗∗^ [0.26, 0.52]	-0.33⁣^∗∗∗^ [-0.47, -0.19]	-0.05 [-0.20, 0.11]	0.13 [-0.03, 0.28]	0.43⁣^∗∗∗^ [0.29, 0.55]	—	0.22⁣^∗∗∗^ [0.13, 0.30]	-0.24⁣^∗∗∗^ [-0.32, -0.16]	-0.18⁣^∗∗∗^ [-0.26, -0.09]
Problem-solving (7)	0.39⁣^∗∗∗^ [0.25, 0.52]	0.18⁣^∗^ [0.02, 0.32]	-0.06 [-0.22, 0.09]	0.05 [-0.11, 0.20]	0.33⁣^∗∗∗^ [0.18, 0.46]	0.30⁣^∗∗∗^ [0.15, 0.44]	—	-0.10⁣^∗^ [-0.18, -0.01]	-0.08⁣^∗^ [-0.16, 0.01]
Depression (8)	-0.55⁣^∗∗∗^ [-0.65, -0.44]	0.42⁣^∗∗∗^ [0.28, 0.54]	0.21⁣^∗∗^ [0.06, 0.36]	0.35⁣^∗∗∗^ [0.21, 0.48]	-0.35⁣^∗∗∗^ [-0.48, -0.21]	-0.39⁣^∗∗∗^ [-0.51, -0.25]	-0.28⁣^∗∗∗^ [-0.42, -0.13]	—	0.49⁣^∗∗∗^ [0.43, 0.56]
Anxiety (9)	-0.32⁣^∗∗∗^ [-0.45, -0.17]	0.44⁣^∗∗∗^ [0.30, 0.56]	0.18⁣^∗^ [0.02, 0.33]	0.17⁣^∗^ [0.01, 0.31]	-0.17⁣^∗^ [-0.32, -0.02]	-0.43⁣^∗∗∗^ [-0.55, -0.30]	-0.19⁣^∗^ [-0.33, -0.03]	0.66⁣^∗∗∗^ [0.56, 0.74]	—

*Note.* Following the decomposition of observed correlations, values above the diagonal are within-person associations using *N* = 648 observations. Correlations below the diagonal represent between-person correlations using the 162 participants. Values in square brackets indicate the 95% confidence interval for each correlation. ∗ indicates *p* < 0.05. ∗∗ indicates *p* < 0.01. ∗∗∗ indicates *p* < 0.001.

## Data Availability

Data to replicate the results reported in this paper are available on request through the *Open Science Framework* (https://osf.io/syk25/) following ethical procedures governing the reuse of sensitive data. We require requestors to complete a data-sharing agreement to ensure participant confidentiality.

## Supplementary Materials

Supplementary Materials The supplementary materials include (1) Supplementary Table S1, presenting the mediation models of rumination, avoidance, and suppression predicting depressive symptoms; (2) Supplementary Table S2, with an overview of the mediation models of reappraisal, acceptance, and problem-solving predicting depressive symptoms; (3) Supplementary Table S3, detailing the mediation models of rumination, avoidance, and suppression predicting anxiety symptoms; (4) Supplementary Table S4, demonstrating the mediation models of reappraisal, acceptance, and problem-solving predicting anxiety symptoms; (5) Supplementary Table S5, providing the effects of study condition on emotion regulation in the mediation models; and (6) Supplementary Table S6, containing fixed effect estimates of multilevel growth analyses for emotion regulation strategies.
