# Supplementary material for: Emotion Regulation as a Mechanism of Mindfulness in Individual Cognitive-Behavioral Therapy for Depression and Anxiety Disorders
Source: Depress Anxiety. 2024 Apr 2;2024:9081139. doi: 10.1155/2024/9081139 (PMC11919210; doi:10.1155/2024/9081139)
Supplement: Supplementary Materials — The supplementary materials include (1) Supplementary Table S1, presenting the mediation models of rumination, avoidance, and suppression predicting depressive symptoms; (2) Supplementary Table S2, with an overview of the mediation models of reappraisal, acceptance, and problem-solving predicting depressive symptoms; (3) Supplementary Table S3, detailing the mediation models of rumination, avoidance, and suppression predicting anxiety symptoms; (4) Supplementary Table S4, demonstrating the mediation models of reappraisal, acceptance, and problem-solving predicting anxiety symptoms; (5) Supplementary Table S5, providing the effects of study condition on emotion regulation in the mediation models; and (6) Supplementary Table S6, containing fixed effect estimates of multilevel growth analyses for emotion regulation strategies. [file 9081139.f1.pdf]

Supplementary Material

for

Emotion Regulation as a Mechanism of Mindfulness in Individual Cognitive-Behavioral Therapy for Depression and Anxiety Disorders

Luise Pruessner[1], Christina Timm[1], Julia Kalmar[2], Hinrich Bents[2], Sven Barnow[1], Johannes Mander[2]

[1] Department of Psychology, Heidelberg University (Germany)

[2] Center for Psychological Psychotherapy, Heidelberg University (Germany)

Author Note

Luise Pruessner https://orcid.org/0000-0002-4796-9682

Christina Timm https://orcid.org/0000-0002-3675-6163

Julia Kalmar https://orcid.org/0000-0002-3952-9070

Sven Barnow https://orcid.org/0009-0004-5359-9384

Johannes Mander https://orcid.org/0000-0003-0358-1106

The authors declare that they have no conflict of interest regarding the publication of this article. Data to replicate the results reported in this paper are available on request through the *Open Science Framework* (https://osf.io/syk25/). Correspondence should be addressed to Luise Pruessner, Department of Psychology, Heidelberg University, Hauptstr. 47-51, 69117 Heidelberg, Germany. Email: luise.pruessner@psychologie.uni-heidelberg.de.

Table S1

Multilevel Structural Equation Modeling with Coefficients for Total, Direct, and Indirect Effects of Rumination, Avoidance, and Suppression on Depressive Symptoms

	Level 1 (*within*)						Level 2 (*between*)					
	Estimate	95% CI		*SE*	*z*	*p*	*Estimate*	95% CI		*SE*	*z*	*p*
Total effect (c)												
Mindfulness → Depression	-11.52	-13.75	-9.30	1.14	-10.14	< .001	-15.46	-19.31	-11.61	1.96	-7.87	< .001
Indirect effects (ab)												
Mindfulness → Rumination → Depression	-1.09	-1.80	-0.39	0.36	-3.03	.002	-2.81	-4.85	-0.78	1.04	-2.71	.007
Mindfulness → Avoidance → Depression	-0.06	-0.55	0.43	0.25	-0.25	.803	0.28	-1.29	1.85	0.80	0.35	.725
Mindfulness → Suppression → Depression	-0.27	-0.63	0.09	0.18	-1.47	.142	-2.48	-4.40	-0.57	0.98	-2.55	.011
Direct effects (c', a, b)												
Mindfulness → Depression	-10.10	-12.43	-7.77	1.19	-8.50	< .001	-10.44	-14.58	-6.30	2.11	-4.94	< .001
Rumination → Depression	1.26	0.53	2.00	0.37	3.39	<.001	4.83	2.76	6.91	1.06	4.57	< .001
Avoidance → Depression	0.10	-0.65	0.84	0.38	0.25	.803	-0.49	-3.21	2.23	1.39	-0.35	.723
Suppression → Depression	0.76	-0.17	1.68	0.47	1.61	.108	4.39	1.78	7.00	1.33	3.30	< .001
Mindfulness → Rumination	-0.86	-1.12	-0.61	0.13	-6.65	< .001	-0.58	-0.93	-0.24	0.18	-3.29	<.001
Mindfulness → Avoidance	-0.65	-0.93	-0.38	0.14	-4.72	< .001	-0.57	-0.85	-0.28	0.15	-3.88	< .001
Mindfulness → Suppression	-0.36	-0.55	-0.16	0.10	-3.57	< .001	-0.57	-0.88	-0.27	0.16	-3.66	< .001

	AIC	BIC	CFI	RMSEA	TLI	SRMR	
Model fit	7429.333	7577.675	0.999	0.031	0.967	0.000	0.014

Note. AIC = Akaike information criterion; BIC = Bayesian information criterion; RMSEA = Root-mean-square error of approximation; CFI = Comparative fit index; TLI = Tucker-Lewis-Index; SRMR = Standardized root-mean-square residual.

Table S2

Multilevel Structural Equation Modeling with Coefficients for Total, Direct and Indirect Effects of Reappraisal, Acceptance, and Problem-Solving on Depressive Symptoms

	Level 1 (*within*)						Level 2 (*between*)					
	Estimate	95% CI		*SE*	*z*	*p*	*Estimate*	95% CI		*SE*	*z*	*p*
Total effect (c)												
Mindfulness → Depression	-11.53	-13.76	-9.30	1.14	-10.14	< .001	-15.37	-19.22	-11.52	1.96	-7.83	< .001
Indirect effects (ab)												
Mindfulness → Reappraisal → Depression	-0.73	-1.38	-0.08	0.33	-2.20	.028	-0.84	-1.62	-0.06	0.40	-2.10	.036
Mindfulness → Acceptance → Depression	-0.70	-1.34	-0.05	0.33	-2.11	.035	-0.59	-1.33	0.15	0.38	-1.55	.120
Mindfulness → Problem-Solving → Depression	0.21	-0.19	0.61	0.21	1.02	.310	0.19	-0.31	0.70	0.26	0.75	.452
Direct effects (c', a, b)												
Mindfulness → Depression	-10.31	-12.64	-7.98	1.19	-8.67	< .001	-10.45	-12.84	-8.06	1.22	-8.57	< .001
Reappraisal → Depression	-1.00	-1.84	-0.17	0.42	-2.36	.018	-1.13	-2.11	-0.15	0.50	-2.26	.024
Acceptance → Depression	-0.90	-1.69	-0.12	0.40	-2.25	.024	-0.75	-1.66	0.16	0.46	-1.61	.107
Problem-Solving → Depression	0.47	-0.41	1.36	0.45	1.05	.293	0.43	-0.67	1.53	0.56	0.77	.444
Mindfulness → Reappraisal	0.73	0.49	0.97	0.12	5.89	< .001	0.74	0.49	0.99	0.13	5.77	< .001
Mindfulness → Acceptance	0.77	0.51	1.03	0.13	5.75	< .001	0.78	0.52	1.05	0.14	5.76	< .001
Mindfulness → Problem-Solving	0.44	0.23	0.66	0.11	4.06	< .001	0.74	0.49	0.99	0.13	5.77	< .001

Model fit	AIC	BIC	CFI	RMSEA	TLI	SRMR	
	7358.585	7507.063	0.997	0.044	0.939	0.000	0.019

Note. AIC = Akaike information criterion; BIC = Bayesian information criterion; RMSEA = Root-mean-square error of approximation; CFI = Comparative fit index; TLI = Tucker-Lewis-Index; SRMR = Standardized root-mean-square residual.

Table S3

Multilevel Structural Equation Modeling with Coefficients for Total, Direct, and Indirect Effects of Rumination, Avoidance, and Suppression on Anxiety Symptoms

	Level 1 (*within*)						Level 2 (*between*)					
	Estimate	95% CI		*SE*	*z*	*p*	*Estimate*	95% CI		*SE*	*z*	*p*
Total effect (c)												
Mindfulness → Anxiety	-7.25	-10.05	-4.45	1.30	-5.59	< .001	-7.87	-11.72	-4.01	1.97	-4.00	< .001
Indirect effects (ab)												
Mindfulness → Rumination → Anxiety	-0.66	-1.38	0.06	0.37	-1.79	.074	-3.05	-5.24	-0.86	1.12	-2.73	.006
Mindfulness → Avoidance → Anxiety	-0.60	-1.18	-0.01	0.30	-2.00	.045	-0.27	-1.86	1.32	0.81	-0.33	.742
Mindfulness → Suppression → Anxiety	0.07	-0.28	0.42	0.18	0.40	.689	-1.06	-2.63	0.51	0.80	-1.32	.186
Direct effects (c’, a, b)												
Mindfulness → Anxiety	-6.07	-8.73	-3.40	1.36	-4.46	< .001	-3.49	-7.64	0.67	2.12	-1.65	.100
Rumination → Anxiety	0.76	-0.05	1.56	0.41	1.85	.065	5.41	3.33	7.48	1.06	5.11	< .001
Avoidance → Anxiety	0.91	0.10	1.72	0.41	2.21	.027	0.46	-2.29	3.21	1.40	0.33	.741
Suppression → Anxiety	-0.20	-1.18	0.78	0.50	-0.40	.687	1.88	-0.76	4.51	1.34	1.40	.163
Mindfulness → Rumination	-0.87	-1.12	-0.62	0.13	-6.73	< .001	-0.56	-0.91	-0.21	0.18	-3.16	.002
Mindfulness → Avoidance	-0.65	-0.93	-0.38	0.14	-4.71	< .001	-0.58	-0.88	-0.27	0.16	-3.68	< .001
Mindfulness → Suppression	-0.36	-0.55	-0.16	0.10	-3.56	< .001	-0.57	-0.85	-0.28	0.15	-3.87	< .001
Model fit	AIC	BIC		CFI			RMSEA		TLI		SRMR	
	7336.274	7488.854		0.990			0.035		0.935		0.027	0.030

Note. AIC = Akaike information criterion; BIC = Bayesian information criterion; RMSEA = Root-mean-square error of approximation; CFI = Comparative fit index; TLI = Tucker-Lewis-Index; SRMR = Standardized root-mean-square residual.

Table S4

Multilevel Structural Equation Modeling with Coefficients for Total, Direct and Indirect Effects of Reappraisal, Acceptance, and Problem-Solving on Anxiety Symptoms

	Level 1 (*within*)						Level 2 (*between*)					
	Estimate	95% CI		*SE*	*z*	*p*	*Estimate*	95% CI		*SE*	*z*	*p*
Total effect (c)												
Mindfulness → Anxiety	-7.25	-9.78	-4.71	1.29	-5.60	< .001	-8.08	-11.90	-4.25	1.95	-4.13	< .001
Indirect effects (ab)												
Mindfulness → Reappraisal → Anxiety	-0.61	-1.31	0.08	0.35	-1.73	.083	1.19	-0.97	3.36	1.11	1.08	.280
Mindfulness → Acceptance → Anxiety	-0.57	-1.26	0.13	0.35	-1.60	.111	-6.12	-9.37	-2.87	1.66	-3.69	< .001
Mindfulness → Problem-Solving → Anxiety	-0.11	-0.54	0.32	0.22	-0.50	.617	-0.08	-1.93	1.76	0.94	-0.09	.931
Direct effects (c’, a, b)												
Mindfulness → Anxiety	-5.96	-8.62	-3.30	1.36	-4.39	< .001	-3.07	-7.81	1.67	2.42	-1.27	.205
Reappraisal → Anxiety	-0.84	-1.75	0.07	0.46	-1.81	.071	1.70	-1.29	4.68	1.52	1.11	.265
Acceptance → Anxiety	-0.74	-1.61	0.13	0.44	-1.66	.098	-7.74	-10.75	-4.72	1.54	-5.03	< .001
Problem-Solving → Anxiety	-0.25	-1.20	0.71	0.49	-0.50	.614	-0.12	-2.82	2.58	1.38	-0.09	.931
Mindfulness → Reappraisal	0.73	0.49	0.97	0.12	5.91	< .001	0.70	0.42	0.99	0.15	4.84	< .001
Mindfulness → Acceptance	0.77	0.51	1.03	0.13	5.74	< .001	0.79	0.50	1.08	0.15	5.30	< .001
Mindfulness → Problem-Solving	0.44	0.23	0.66	0.11	4.06	< .001	0.68	0.40	0.96	0.14	4.81	< .001

	AIC	BIC	CFI	RMSEA	TLI	SRMR	
Model fit	7448.875	7597.353	0.999	0.018	0.987	0.000	0.016

Note. AIC = Akaike information criterion; BIC = Bayesian information criterion; RMSEA = Root-mean-square error of approximation; CFI = Comparative fit index; TLI = Tucker-Lewis-Index; SRMR = Standardized root-mean-square residual.

Table S5

Multilevel Structural Equation Modeling with Effects of Study Condition on Emotion Regulation

Model	*Estimate*	[95% CI]	*SE*	*z*	*p*
Depressive Symptoms					
Condition → Rumination	0.13	[0.13; 0.96]	0.336	-0.13	.394
Condition → Avoidance	0.12	[0.12; 0.98]	0.329	-0.12	.353
Condition → Suppression	0.03	[0.11; 0.23]	0.820	-0.19	.243
Condition → Reappraisal	0.02	[-0.20; 0.23]	0.11	0.17	.868
Condition → Acceptance	-0.05	[-0.27; 0.18]	0.11	-0.41	.682
Condition → Problem-Solving	-0.10	[-0.31; 0.11]	0.11	-0.93	.354
Anxiety Symptoms					
Condition → Rumination	0.13	[-0.14; 0.39]	0.14	0.93	.353
Condition → Avoidance	0.12	[-0.12; 0.35]	0.12	0.97	.332
Condition → Suppression	0.02	[-0.20; 0.24]	0.11	0.18	.855
Condition → Reappraisal	0.03	[-0.19; 0.24]	0.11	0.25	.806
Condition → Acceptance	-0.06	[-0.27; 0.16]	0.11	-0.50	.617
Condition → Problem-Solving	-0.10	[-0.31; 0.11]	0.11	-0.95	.342

Note. For the MSEM analyses, the study condition was used as a between-person predictor (level 2).

Table S6

Fixed Effect Estimates and Model Fit of Multilevel Growth Analyses for Emotion Regulation Strategies

Predictor	Rumination		Avoidance		Suppression		Reappraisal		Acceptance		Problem-Solving	
	b [95% CI]	*p*	*b* [95% CI]	*p*	*b* [95% CI]	*p*	*b* [95% CI]	*p*	*b* [95% CI]	*p*	*b* [95% CI]	*p*
(Intercept)	5.45***<br>[4.74; 6.16]		2.96***<br>[2.29; 3.62]		2.73***<br>[2.13; 3.33]		2.34***<br>[1.68, 3.00]		2.26***<br>[1.59, 2.94]		3.67***<br>[3.05; 4.28]	
Age	-0.01<br>[-0.02; 0.00]	.085	0.01<br>[0.00; 0.02]	.115	0.01<br>[0.00; 0.02]	.108	0.00<br>[-0.01, 0.01]	.701	0.00<br>[-0.01, 0.01]	.717	0.00<br>[-0.01; 0.01]	.657
Gender	-0.49***<br>[-0.75, -0.23]	.001	0.08<br>[-0.16; 0.31]	.504	0.16<br>[-0.06; 0.37]	.157	0.03<br>[-0.21, 0.27]	.814	0.27*<br>[0.03, 0.51]	.028	-0.05<br>[-0.27; 0.17]	.664
Diagnosis	-0.08<br>[-0.33; 0.17]	.543	0.25*<br>[0.02; 0.48]	.031	-0.02<br>[-0.24; 0.19]	.848	-0.13<br>[-0.37, 0.10]	.260	-0.19<br>[-0.43, 0.04]	.105	-0.19<br>[-0.41; 0.03]	.082
Time	-0.23***<br>[-0.31, -0.14]	.001	-0.12**<br>[-0.22, -0.03]	.008	-0.08*<br>[-0.15, -0.01]	.018	0.14**<br>[0.06, 0.22]	.001	0.18***<br>[0.09, 0.27]	.001	0.11**<br>[0.03; 0.18]	.005
Condition<br>(TAU + M vs. TAU)	-0.26<br>[-0.68; 0.16]	.223	0.07<br>[-0.35; 0.49]	.754	0.01<br>[-0.34; 0.35]	.972	0.02<br>[-0.38, 0.42]	.919	0.16<br>[-0.25, 0.58]	.439	0.45*<br>[0.08; 0.81]	.015
Condition<br>(TAU + M vs. TAU + PMR)	-0.24<br>[-0.66; 0.18]	.252	-0.12<br>[-0.54; 0.30]	.578	0.00<br>[-0.34; 0.34]	.999	0.02<br>[-0.38, 0.42]	.914	0.26<br>[-0.15, 0.68]	.214	0.23<br>[-0.13; 0.60]	.201
Time × Condition<br>(TAU + M vs. TAU + PMR)	0.06<br>[-0.06; 0.17]	.355	-0.09<br>[-0.21; 0.04]	.179	-0.03<br>[-0.12; 0.06]	.561	0.00<br>[-0.12, 0.11]	.988	-0.05<br>[-0.17, 0.08]	.461	-0.11*<br>[-0.22, -0.01]	.029
Time × Condition<br>(TAU + M vs. TAU + PMR)	0.04<br>[-0.08; 0.16]	.508	0.01<br>[-0.12; 0.14]	.898	-0.02<br>[-0.11; 0.08]	.743	0.01<br>[-0.11, 0.13]	.842	-0.05<br>[-0.17, 0.08]	.475	-0.04<br>[-0.15; 0.06]	.416
AIC	1292.48		1315.00		1063.33		1249.02		1307.20		1140.59	
BIC	1339.48		1361.92		1110.33		1296.02		1354.21		1187.58	
Pseudo-R^2 (fixed effects)	0.12		0.06		0.04		0.04		0.06		0.03	
Pseudo-R^2 (total)	0.63		0.50		0.65		0.57		0.55		0.59	

Note. AIC = Akaike information criterion; BIC = Bayesian information criterion. * indicates $p < .05$. ** indicates $p < .01$. *** indicates $p < .001$.
